# Is there an overestimation of dengue compared with that of other acute febrile syndromes in childhood?

**DOI:** 10.1371/journal.pntd.0012137

**Published:** 2024-06-07

**Authors:** Sônia Maria Cavalcante da Rocha, Raull Costa Pires, Daniela Cristina Sensato Monteiro, Thaís Cavalcante Rocha Cronemberges, Natália Vasconcelos de Souza, Jeová Keny Baima Colares, Danielle Malta Lima

**Affiliations:** 1 University of Fortaleza (UNIFOR), Graduate Program in Medical Sciences, Fortaleza, Ceará, Brazil; 2 Hospital Infantil Albert Sabin, Fortaleza, Ceará, Brazil; 3 University of Fortaleza (UNIFOR), Fortaleza, Ceará, Brazil; 4 University of Fortaleza (UNIFOR/RENORBIO), Postgraduate Program in Biotechnology, Ceará State University, Fortaleza, Ceará, Brazil; Public Health Agency of Canada, CANADA

## Abstract

A group of children with clinical suspicion of dengue were assessed to determine if there was an overestimation of dengue compared with that of leptospirosis and leishmaniasis. This descriptive and analytical cross-sectional study, based on the active search of participants with acute febrile illness, was conducted at two pediatric hospitals. The collection of clinical and epidemiological data was performed using questionnaires, and laboratory tests specific for dengue were performed using immunochromatographic, serological, and molecular methods. Dengue-negative samples were assessed for *Leptospira* and *Leishmania* spp. using molecular tests. Data were assessed using analysis of variance (ANOVA), the chi-square test, and Fisher’s exact test. In total, 86 participants were evaluated, of whom 39 (45%) were positive for dengue fever, 4 (5%) for leptospirosis, and 1 (1%) for leishmaniasis. Forty-two participants (49%) presented dengue-like symptoms. The predominant age range for the virus was 3–10 years. Most clinical manifestations were nonspecific, with frequent concomitant gastrointestinal and respiratory symptoms. Furthermore, we found that the acute febrile syndrome in childhood persists as a challenge for health professionals, especially in the early days of the disease, due to a plurality of diagnostic hypotheses, associated with the difficulty of establishing well-defined symptoms in children, especially in infants. Dengue fever continues to be a frequent pathology with acute febrile infections in childhood; however, there is an overestimation of the disease, especially in endemic regions, when one considers only the clinical epidemiological diagnosis.

## Author summary

Dengue is a viral disease present in over 100 countries that is transmitted via mosquito bites. Most cases of dengue are associated with mild symptoms, with some cases leading to severe complications and even death. The clinical diagnosis of dengue is challenging because it presents with nonspecific symptoms that can be confused with those of other diseases, and specific laboratory tests are required for confirmation. In this study, children and adolescents who experienced a fever for 24 h to 10 d and had a clinical suspicion of dengue were evaluated and tested using specific laboratory methods. Negative samples were analyzed for leptospirosis and leishmaniasis because, despite being distinct diseases, they share certain clinical characteristics, such as different fever forms. Of the 86 children evaluated in this study, 39 were positive for dengue (45%), four for leptospirosis (5%), and one for leishmaniasis (1%). Therefore, a specific laboratory diagnosis is important, especially in children, to minimize morbidity and mortality.

## Introduction

Fever is one of the most common causes for medical consultations with pediatric emergency services [1]. Typically, a fever is accompanied by nonspecific symptoms, thereby making it difficult to diagnose and treat [2]. The presence of a fever combined with nonspecific clinical manifestations for a period exceeding 24 h and less than 7 d is called acute undifferentiated febrile illness, and infectious agents, whether viral or bacterial, are the main agents that trigger the clinical presentation [3]. Infections belonging to this group include rubella, dengue fever, measles, enteroviruses, influenza, chikungunya, viral hepatitis, yellow fever, leptospirosis, meningococcemia, and meningitis [4].

Due to the symptoms associated with dengue fever, other illnesses such as leptospirosis are often misdiagnosed as dengue fever, thereby leading to an overestimation of their prevalence. Therefore, the prevalence of dengue fever within a study population was investigated. Taking the study area into consideration, Fortaleza, Ceará, Brazil, other similar diseases such as leptospirosis was compared to dengue [5]. Additionally, the presence of visceral leishmaniasis was also investigated as it shares similar characteristics to dengue and leptospirosis during its initial or oligosymptomatic phase [6,7], thus, visceral leishmaniasis was also included in this study [8]. Therefore, this study aimed to evaluate a group of children in the Fortaleza area with clinically suspected dengue to determine whether there was an overestimation of dengue compared with leptospirosis and leishmaniasis.

## Methods

### Ethics statement

This study was approved by the Research Ethics Committee of the Hospital Infantil Albert Sabin, Fortaleza (CAAE: 45741415.1.0000.5042). Participation was voluntary and consent forms were read and signed on behalf of the parent or guardian of any child or adolescent.

Between September 2015 to March 2016, a total of 86 participants who adhered to the selection criteria were included in this study. Participants with undifferentiated acute febrile syndrome were prospectively recruited from two pediatric hospitals in the city of Fortaleza by actively searching outpatient clinics and wards according to the following inclusion criteria: children (aged 0–12 years) and adolescents (up to 18 years) presenting a fever lasting more than 24 h and less than 10 d, associated with at least one of the following symptoms: headache, retroorbital pain, arthralgia, myalgia, exanthema, and prostration. The exclusion criteria included the presence of comorbidities (previous heart disease, diabetes mellitus, and hemoglobinopathies), clinical manifestations suggesting an alternative diagnosis (pneumonia, otitis, etc.), and contraindications to blood sample collection. According to the Ministry of Health, dengue is an acute febrile illness accompanied by at least two of the following symptoms: headache, retroorbital pain, myalgia, arthralgia, prostration, and/or rash.

Clinical data were obtained through a questionnaire administered during medical care and collection of peripheral blood. These samples were used for nonspecific tests, such as hematological and biochemical parameters, and specific diagnostic tests (immunochromatographic, serological, and molecular tests).

A protocol was established to conduct molecular and serological tests, in which all participants were tested initially for dengue through tests Dengue NS1Ag STRIP immunochromatographic test (Bio-Rad Laboratories), Platelia Dengue NS1 Ag-ELISA (Bio-Rad Laboratories), Dengue IgM-ELISA (Bioeasy Diagnostica Ltda), and reverse transcription polymerase chain reaction (RT-PCR) using specific primers as stated by Henchal et al. [9]. Samples with negative results were tested for Leptospira and Leishmania spp. by PCR, using specific primers as presented in Gravekamp et al., and Degrave et al., [10,11].

The mean values of stratification by diagnosis and age were analyzed using a nonparametric analysis of variance (ANOVA) with Tukey’s post-hoc test. Categorical variables were evaluated using the chi-squared or Fisher’s exact tests. The level of significance used was 95%. Statistical significance was set at p < 0.05.

## Results

Regarding the distribution of the clinical manifestations, prostration was the most common symptom affecting 73%, and pleural effusion, ecchymosis, hematuria, and hypothermia were less prevalent, all affecting 1% of the total population sample (N = 86).

Another important finding was the frequent presence of respiratory and gastrointestinal manifestations in the studied population (N = 86), with vomiting, cough, and rhinorrhea the most observed symptoms (30%, 27%, and 25%, respectively).

Of the 86 participants examined, 39 (45%) of the confirmed cases had dengue; of these, 1 (2.5%) was positive for dengue by the Platelia Dengue NS1 Ag-ELISA, 37 (94.8%) by RT-PCR, and 3 (7.6%) by Dengue IgM-ELISA. In addition, 5% of the patients had leptospirosis, 1% had leishmaniasis, and 49% had negative results for these diseases; the latter group was referred to as dengue-like disease. The positive patient in the Platelia Dengue NS1-Ag-ELISA test had 4 days of fever. The 3 positive patients in the Elisa IgM test for dengue had 6 days of fever and the positive patients in the RT-PCR ranged from 1–9 days of fever. A greater number of positive cases were observed from the 1st to 3rd day via RT-PCR. The NS1Ag STRIP immunochromatographic test, based on the detection of glycoprotein NS1, showed negative results in all blood samples. The ELISA-NS1 and ELISA IgM tests showed low positivity, both at 2.5%.

When correlating general manifestations with pathologies, retro-orbital pain and myalgia were frequent in children diagnosed with dengue (66.7% and 63.3%, respectively), whereas arthralgia (54,5%) and exanthema (51.9%) were usually present in children with dengue-like disease (Table 1).

**Table 1:** Association of clinical parameters observed in patients with dengue, leptospirosis, and dengue-like diseases.

General Clinical Manifestations	Diagnosis			p^a^ Value
	Dengue	Leptospirosis	Dengue-like disease	
**Headache**	25 (48.1%)	3 (5.8%)	24 (46.2%)	0.764
**Retro-Orbital Pain**	10 (66.7%)	1 (6.7%)	4 (26.7%)	0.155
**Myalgia**	19 (63.3%)	1 (3.3%)	10 (33.3%)	0.063
**Arthralgia**	5 (45.5%)	-	6 (54.5%)	0.536
**Prostration**	30 (48.4%)	2 (3.2%)	30 (48.4%)	0.481
**Exanthema**	11 (40.7%)	2 (7.4%)	14 (51.9%)	0.653
**Abdominal Pain**	8 (66.7%)	-	4 (33.3%)	0.223
**Low Blood Pressure**	2 (50%)	-	2 (50%)	0.816
**Drowsiness**	6 (75%)	-	2 (25%)	0.195
**Dehydration**	10 (52.6%)	1 (5.3%)	8 (42.1%)	0.832
**Rhinorrhea**	9 (42.9%)	2 (9.5%)	10 (47.6%)	0.557
**Cough**	10 (43.5%)	2 (8.7%)	11 (47.8%)	0.631
**Dyspnea**	3 (50%)	-	3 (50%)	0.730
**Sore Throat**	7 (53.8%)	-	6 (46.2%)	0.453
**Nauseas**	9 (42.9%)	2 (9.5%)	10 (47.6%)	0.724
**Vomit**	13 (52%)	1 (4%)	11 (44%)	0.830
**Diarrhea**	9 (52.9%)	-	8 (47.1%)	0.369

^a^ Chi-Square Test

*p<0.05.

Severe manifestations include abdominal pain and drowsiness, which were frequently observed in individuals with dengue fever (66.7% and 75%, respectively), and dengue-like disease (33.3% and 25%, respectively) (Table 1). Hematological data at the time of consultation showed that the results of hemoglobin and hematocrit in the 3–10 years age group with dengue were significantly (p < 0.05) lower than the normal standard for that age group, whereas the participants aged 0–2 were above the normal percentile for that age.

White blood cell counts (lymphocytes) for participants aged 0–2 (52.9%) and 3–10 (58.8%) years in the dengue-like group were significantly higher (p > 0.05) than the normal range.

The mean platelet count was significantly lower in the dengue- and dengue-like groups than in the leptospirosis group.

The following profile was obtained from a patient diagnosed with leishmaniasis: female, 4 years of age, fever for 5 days, prostration, headache, myalgia, rhinorrhea, vomiting, and epistaxis. The hemogram showed anemia (hematocrit: 28.9% and hemoglobin: 9.2 mg/dL), leucopenia (3.500/mm3) and thrombocytopenia (88.000/mm3); urea: 20 mg/dL; creatinine: 0.6 mg/dL; albumin: 3.2 mg/dL; AST: 15 mg/dL; alanine aminotransferase (ALT): 71 mg/dL. Both Kalazar Detect (InBios International, Seattle, WA, USA) (K-39) and PCR for Leishmania spp. were positive.

## Discussion

Dengue has been endemic in the municipality of Fortaleza since 1986, when the dengue 1 serotype was introduced [12]. And the clinical diagnosis of dengue is challenging because it presents with nonspecific symptoms that can be confused with those of other diseases.

In the present study, a high incidence of dengue fever was confirmed, with 45% of patients within the being positive, together with a marked proportion of underreporting of other acute febrile syndromes (49%). It was assumed that the overestimation of dengue compared with that of other febrile syndromes was due to its wide clinical spectrum [13] and the benign and self-limiting nature of most other common childhood pathologies, which due to their low rates of complications, are not investigated with regard to the etiological agent [14].

Similarities were observed when no significant differences (p > 0.05) were found after stratifying the variables for sex, age range, and clinical manifestations. In the case of variable sex, the results can be attributed to the changing recreational profile of children today. Since there are no large disparities in sex-related leisure activities, it is possible to expose them to contaminated water, leading to leptospirosis, or to stay at home due to technological expansion (television, games, Internet, etc.), favoring the vectors of the dengue virus, which act mainly in domicile and peridomicile conditions [15].

With respect to age group, the results corroborate those of other studies that showed no predilection for age group in cases diagnosed with dengue [16]. However, there was a divergence regarding anicteric leptospirosis because, according to Levett, PN [17], older children are more susceptible. This divergence may be associated with the research site, that is, hospitals located in the periphery of the city and those with a high prevalence of care for individuals with poor housing and basic sanitation, favoring the transmission of leptospirosis to all age groups.

In the case of clinical manifestations, two aspects were considered in patients with dengue, leptospirosis, and dengue-like diseases. Initially, in an isolated manner, the following frequencies were evidenced in decreasing order: prostration (72%), headache (60%), myalgia (35%), exanthema (31%), vomiting (29%), and the least prevalent (hypothermia and bleeding, both with approximately 1%) (Table 1). Similar results were observed by Larocque et al. (2005) [18], Bruce et al. (2005) [19], and Libraty et al. (2007) [20] in Bangladesh, Puerto Rico, and Thailand, respectively, when comparing dengue and leptospirosis.

Subsequently, the symptoms were analyzed with respect to severity, following the recommendations of the World Health Organization (WHO) [21] regarding suspected dengue patients. Respiratory (rhinorrhea, cough, and sore throat) and gastrointestinal (nausea, vomiting, and diarrhea) symptoms were sub-classified to evaluate their prevalence in the pediatric population.

The data demonstrated that in childhood, alterations in the respiratory and gastrointestinal systems are common, with the presence of respiratory symptoms being more frequent. Moreover, arthralgia, headache, retro-orbital pain, and myalgia, which are classical manifestations of dengue, can be interpreted as irritability, especially in younger children such as infants.

With respect to nonspecific laboratory test results, the average platelet count was significantly lower in the dengue- and dengue-like groups than in the leptospirosis group. The data indicated that in oligosymptomatic cases, no relevant laboratory abnormalities occurred, validating the findings of other authors who considered the direct relationship between the severity and changes found in the hemogram and hepatic and renal biomarkers [22–24].

Regarding other manifestations with warning signs, two participants older than 11 years were presented with hypotension, and there was a record of pleural effusion. Therefore, in the analyzed population, the proportion of patients requiring hospitalization was not significantly different from the number of attendees. This small number of hospitalizations may have occurred because of the small number of samples analyzed and/or early care in most cases (<5 d of fever).

In contrast to other studies, the results showed no detection of the NS1 glycoprotein. In general, there is consensus among most studies on the high sensitivity and specificity of immunochromatographic tests, especially until the third day of the disease [25,26]. However, Silva et al. [25] argued that the NS1-dengue test may not present reliable results in patients with very low viremia or in those who are not in the first infection and who have been infected by the DENV-2 serotype, because there is a reduction in the sensitivity of NS1 kits due to the previous existence of IgG antibodies that would bind to the antigen, resulting in a non-reactive result. It is assumed that all non-reactive tests in this study were due to several reasons, such as low viremia, previous exposure to the virus, and the possibility that DENV-2 circulation was predominant in the city at the time of the study.

The low percentage for the ELISA-NS1 and ELISA-IgM tests could be attributed to the high number of participants with a febrile period of less than 5 d, since circulating IgM antibody titers are detected mainly after the fifth day of the disease [25,26].

Another fact worthy of note that can possibly explain the high percentage of negative and positive results for dengue-like diseases was that at the time of collection, the national media reported isolated outbreaks of the viral infections chikungunya and zika in neighboring states. Thus, it is possible they had not been diagnosed due to lack of specific kits, given that they have a high clinical-epidemiological and laboratory correspondence with dengue. Furthermore, in many results of this group, elevation of hematocrit and hemoglobin, reduction of segmented and elevation of lymphocytes, and decrease in the platelet count were observed.

A possible limitation of this study was the low study population as it was not possible to perform comparisons between the dengue fever, leptospirosis, and leishmaniasis groups due to a single PCR-positive result for Leishmania. However, despite the reduced sample size, this result is important because the patient was in the first days of the disease (acute phase) and did not have the classic clinical features of infection [27]. However, hematological and epidemiological data justified the assessment, as the hemogram showed pancytopenia, a typical pattern of the disease [28].

Finally, it is important to emphasize that during the survey, one of the participants who had initially been admitted with a clinical diagnosis of dengue with alarm signals, showed a positive result for meningococcemia after the completion of lumbar puncture for the study of cerebrospinal fluid. Meningococcal disease is a major infectious cause of childhood mortality, which may vary from a nonspecific presentation to death of the patient [29]. Thus, considering the severity of the pathology, treatment could have been introduced very early if the molecular methods were more accessible to the public health units.

Another limitation of this study included the description of symptomatology (arthralgia, myalgia, and headache) for the age range of 0–2 years in which the descriptions were provided by the guardians, and these may have been over- or underestimated. Moreover, the percentage of hospitalizations may have been influenced by the exclusion criteria of the study design because patients with comorbidities did not participate in the sample of the population. Consequently, this event may have been responsible for the conflicting results of our study regarding the data supplied by the municipality regarding the number of hospitalizations. In addition, the low number of leishmaniasis-positive participants in the study population hampered the correlation between the variables. Dengue fever continues to be a frequent pathology with acute febrile infections in childhood; however, there is an overestimation of the disease, especially in endemic regions, when one considers only the clinical epidemiological diagnosis.
